# Supplementary material for: A novel RSW&TST framework of MCPs detection for abnormal pattern recognition on large-scale time series and pathological signals in epilepsy
Source: PLoS One. 2021 Dec 22;16(12):e0260110. doi: 10.1371/journal.pone.0260110 (PMC8694460; doi:10.1371/journal.pone.0260110)
Supplement: Attachment — Submitted filename: Response to Reviewers-vf.docx [file pone.0260110.s001.docx]

Please submit your revised manuscript by Oct 25 2021 11:59PM. If you will need more time than this to complete your revisions, please reply to this message or contact the journal office at [plosone@plos.org](mailto:plosone@plos.org). When you're ready to submit your revision, log on to https://www.editorialmanager.com/pone/ and select the 'Submissions Needing Revision' folder to locate your manuscript file.

Please include the following items when submitting your revised manuscript:

- A rebuttal letter that responds to each point raised by the academic editor and reviewer(s). You should upload this letter as a separate file labeled 'Response to Reviewers'.
- A marked-up copy of your manuscript that highlights changes made to the original version. You should upload this as a separate file labeled 'Revised Manuscript with Track Changes'.
- An unmarked version of your revised paper without tracked changes. You should upload this as a separate file labeled 'Manuscript'.

If you would like to make changes to your financial disclosure, please include your updated statement in your cover letter. Guidelines for resubmitting your figure files are available below the reviewer comments at the end of this letter.

If applicable, we recommend that you deposit your laboratory protocols in protocols.io to enhance the reproducibility of your results. Protocols.io assigns your protocol its own identifier (DOI) so that it can be cited independently in the future. For instructions see:<https://journals.plos.org/plosone/s/submission-guidelines#loc-laboratory-protocols>. Additionally, PLOS ONE offers an option for publishing peer-reviewed Lab Protocol articles, which describe protocols hosted on protocols.io. Read more information on sharing protocols at<https://plos.org/protocols?utm_medium=editorial-email&utm_source=authorletters&utm_campaign=protocols>.

We look forward to receiving your revised manuscript.

Kind regards,

Amita Nandal
Academic Editor
PLOS ONE

Journal Requirements:

When submitting your revision, we need you to address these additional requirements.

1. Please ensure that your manuscript meets PLOS ONE's style requirements, including those for file naming. The PLOS ONE style templates can be found at 
https://journals.plos.org/plosone/s/file?id=wjVg/PLOSOne_formatting_sample_main_body.pdf and 
https://journals.plos.org/plosone/s/file?id=ba62/PLOSOne_formatting_sample_title_authors_affiliations.pdf

2. PLOS requires an ORCID iD for the corresponding author in Editorial Manager on papers submitted after December 6th, 2016. Please ensure that you have an ORCID iD and that it is validated in Editorial Manager. To do this, go to ‘Update my Information’ (in the upper left-hand corner of the main menu), and click on the Fetch/Validate link next to the ORCID field. This will take you to the ORCID site and allow you to create a new iD or authenticate a pre-existing iD in Editorial Manager. Please see the following video for instructions on linking an ORCID iD to your Editorial Manager account: <https://www.youtube.com/watch?v=_xcclfuvtxQ>

3. Thank you for stating the following in the Acknowledgments Section of your manuscript:

"I would like to thank Prof. Mohan Karunanithi of the Australia e-Health Research Centre, CSIRO Computation Informatics, for his assistance, support and advice for this paper. This paper is supported by the National Natural Science Foundation of China (No.61104154), and the Specialized Research Fund of the Natural Science Foundation of Shanghai (no.16ZR1401300 and no.16ZR1401200)."

We note that you have provided funding information that is not currently declared in your Funding Statement. However, funding information should not appear in the Acknowledgments section or other areas of your manuscript. We will only publish funding information present in the Funding Statement section of the online submission form. 
Please remove any funding-related text from the manuscript and let us know how you would like to update your Funding Statement. Currently, your Funding Statement reads as follows:

"This paper is supported by National Natural Science Foundation of China (No.61104154), and Specialized Research Fund for Natural Science Foundation of Shanghai (no.16ZR1401300 and no.16ZR1401200)"

Please include your amended statements within your cover letter; we will change the online submission form on your behalf

Response:

We have revised this acknowledgement part by adding another Professor. Qing Zhang to this acknowledgment, and then removing the funding-related text from the manuscript.

4. Please include captions for your Supporting Information files at the end of your manuscript, and update any in-text citations to match accordingly. Please see our Supporting Information guidelines for more information: http://journals.plos.org/plosone/s/supporting-information.

Response:

We have revised the parts of “Supporting Information” at the end of the manuscript, and update the in-text citations to match accordingly.

[Note: HTML markup is below. Please do not edit.]

Reviewers' comments:

Reviewer's Responses to Questions

**Comments to the Author**

1. Is the manuscript technically sound, and do the data support the conclusions?

The manuscript must describe a technically sound piece of scientific research with data that supports the conclusions. Experiments must have been conducted rigorously, with appropriate controls, replication, and sample sizes. The conclusions must be drawn appropriately based on the data presented.

Reviewer #1: Yes

Reviewer #2: Partly

2. Has the statistical analysis been performed appropriately and rigorously?

Reviewer #1: Yes

Reviewer #2: Yes

3. Have the authors made all data underlying the findings in their manuscript fully available?

The [PLOS Data policy](http://www.plosone.org/static/policies.action#sharing) requires authors to make all data underlying the findings described in their manuscript fully available without restriction, with rare exception (please refer to the Data Availability Statement in the manuscript PDF file). The data should be provided as part of the manuscript or its supporting information, or deposited to a public repository. For example, in addition to summary statistics, the data points behind means, medians and variance measures should be available. If there are restrictions on publicly sharing data—e.g. participant privacy or use of data from a third party—those must be specified.

Reviewer #1: Yes

Reviewer #2: Yes

4. Is the manuscript presented in an intelligible fashion and written in standard English?

PLOS ONE does not copyedit accepted manuscripts, so the language in submitted articles must be clear, correct, and unambiguous. Any typographical or grammatical errors should be corrected at revision, so please note any specific errors here.

Reviewer #1: Yes

Reviewer #2: Yes

5. Review Comments to the Author
Please use the space provided to explain your answers to the questions above. You may also include additional comments for the author, including concerns about dual publication, research ethics, or publication ethics. (Please upload your review as an attachment if it exceeds 20,000 characters)

Reviewer #1: 1. State of the art technologies and algorithms achieve very good results.
2. Figure quality is not clear so try to improve it.
3. Proofread the manuscript for grammatical errors.
4. The readability, presentation and organization of this manuscript can be improved.

Response:

(1) We have reedited and improved the quality of Figures.
(2) Including the corresponding author and all coauthors, we have adjusted the presentation and organization in the revised manuscript,
(3) and then proofread the manuscript for grammatical errors, and improved the readability as we can do.

Reviewer #2: 1. The paper is not properly structured.
2. Proposed methodology section is not present. Instead the methods available in literature along with the proposed method is presented in Section 2.
3. It is strongly advised to restructure the paper by separating Background section and proposed method section.
4. Results section needs improvement. Include more comparison results with latest methods.

Response:

According to the suggestions of reviewer, we have，

(1) restructured the paper by separating Background section and proposed method section in the revised version,
(2) introduced another WBS&CUSUM method for MCPs detection, and added the related contents in the methodology part.
(3) and then, improved the result section by adding the comparison results of our RSW&TST with the WBS&CUSUM method, in the synthetic simulations and experiments on real pathological ECG datasets.
